# Supplementary material for: Inorganic Arsenic Induces Elevated p53 Levels with Altered Functionality Impacting the Expression of Toll-like Receptor 3 and Other Target Genes in Immortalized Prostate Epithelial Cells
Source: Int J Mol Sci. 2025 Apr 29;26(9):4253. doi: 10.3390/ijms26094253 (PMC12072582; doi:10.3390/ijms26094253)
Supplement: Supplementary file 1 [file ijms-26-04253-s001.zip › ijms-3545533-supplementary.pdf]

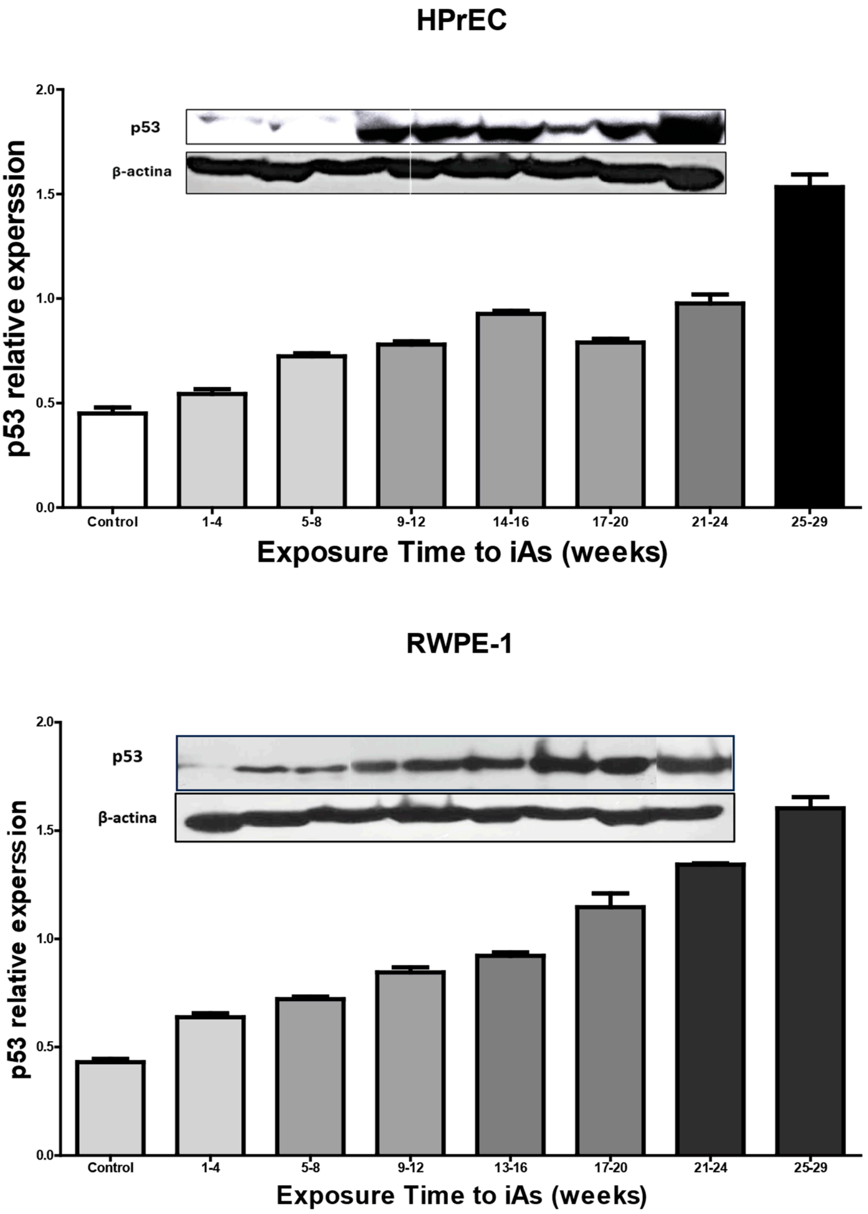

Supplementary Figure S1. Effect of iAs on p53 Expression after 29 Weeks of Toxic Exposure.

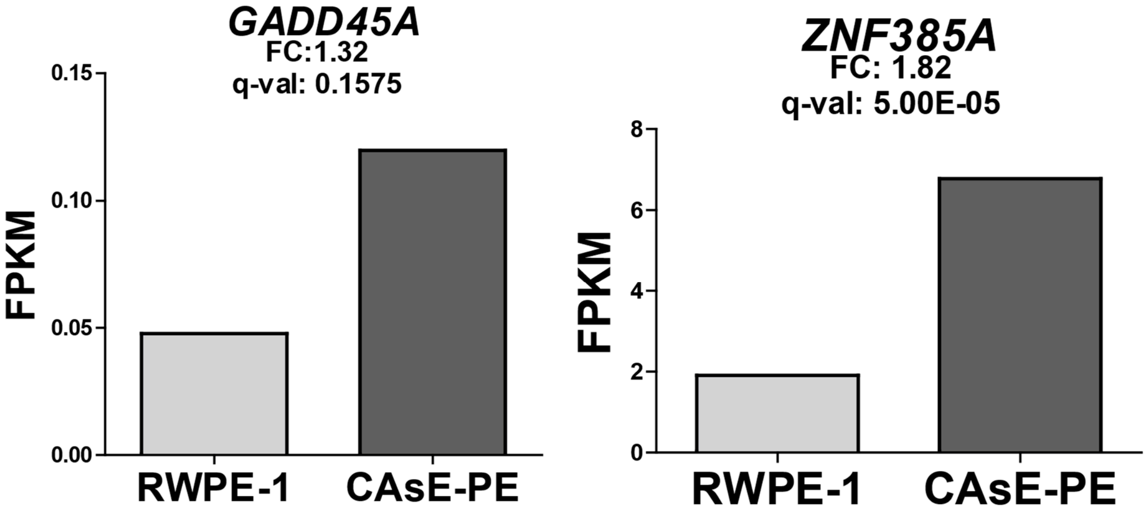

Supplementary Figure S2. Effect of iAs on the expression of p53 target genes GADD45A and *ZNF385* in RWPE-1 and CAsE-PE cells. RNA-seq data from RWPE-1 and CAsE-PE cells (Merrick et al. 2019). FPKM (Fragments Per Kilobase of Exon per Million Reads), FC: log2 (Fold Change), representing the change in gene expression; q-val: adjusted p-value.

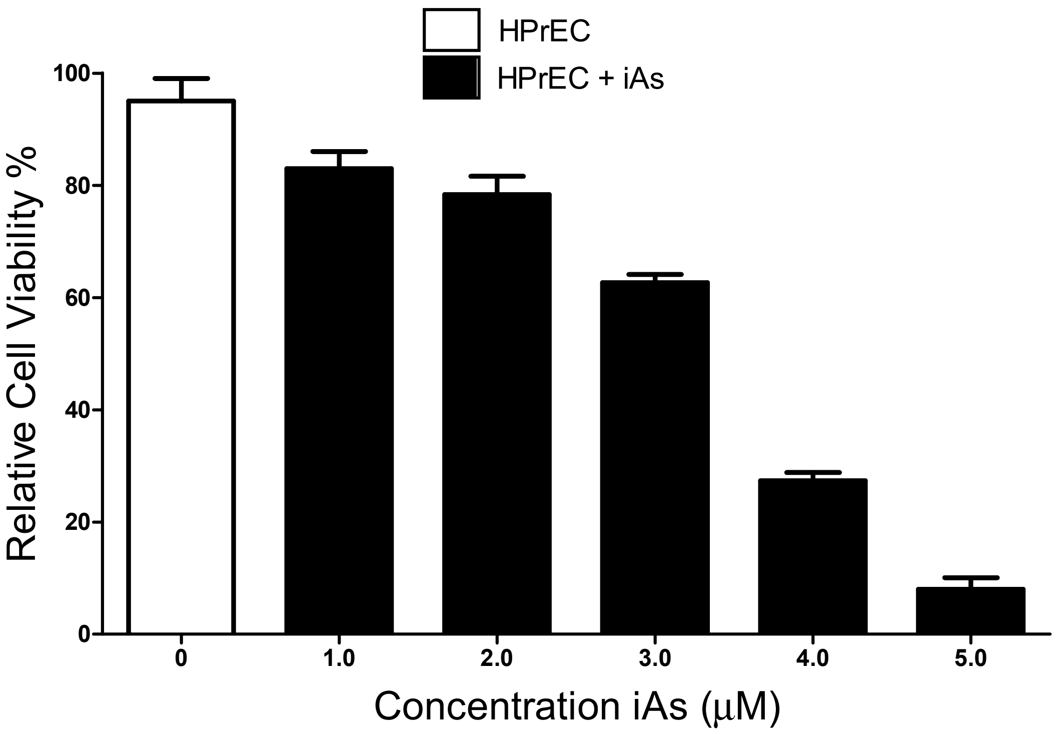

Supplementary Figure S3. Effect of increasing concentrations of sodium arsenite (NaAsO) on prostate epithelial cell viability. A progressive decrease in cell viability was observed with increasing concentrations of iAs. At 3.0 μM, viability dropped below 60%, indicating a significant cytotoxic effect. This concentration was selected for long-term (29-week) exposure experiments, following the transformation model originally described by Tokar et al. (2010), in which RWPE-1 cells were exposed to iAs to generate the CAsE-PE cell line.

Supplementary Table S1. Oligonucleotides Sequences for q-PCR.

**Gene**	Sequence	Alignment Temperature (TA)
hTLR3	Fw: 5'-CACCACCAGCAATACAA C-3'	48°C
	Rv: 3'-GTGTAAGGGAGAATGAG C-5'	
hTP53	Fw: 5'-CTCAAAAGTCTAGAGCCAC C-3'	49°C
	Rv: 3'-TCCCAGAATGCAAGAAG C-5'	
HDMX	Fw: 5'-TCTGACAGTGCTTGCAGG A-3'	52°C
	Rv: 3'-AACATTTCACCTTGCGCAC C-5'	
GAPDH	Fw: 5'-AAGGTGAAGGTCGGAGTCA A-3'	50°C
	Rv: 3'-AATGAAGGGGTCATTGATG G-5'	

Supplementary Table S2. Oligonucleotides sequences used for ChIP/q-PCR.

Gene	Sequence	Alignment Temperature (TA)
hTLR3	Fw 5'-GCACTCATGACTCAATGCAAA C-3'	57°C
	Rv 3'-CACTTTAGGAGATTGCCTTGAG C-5'	
hCDKN1A	Fw 5'-CCAGCCCTTTGGATGGTT T-3'	60°
	Rv 3'-GCCTCCTTTCTGTGCCTG A-5'	

Supplementary Table S3. Sequences of the forward and reverse primers used for PCR amplification of TP53 domains.

Primer	Sequence	Alignment Temperature (TM)
N-p53_p28F	Fw 5'- GGGGGATCCATGGAGGAGCCGCA G-3'	60°C
N-p53_p28R	Rv 3'-CCCTCAACGGAAACCGTATTCGA A-5'	
DBD-P53-P28_F	Fw 5'- AACGGATCCATGCTGGGCTTCTT -3'	60°C
DBD-P53-P28_R	Rv 3'- CCCAAGCTTTCATTCCTCTGTGCGC -5'	
C-P53-P28_F	Fw 5'- AAGGGATCCATGGAGAATCTCCGCAAGAAAG -3'	63°C
C-P53-P28_R	Rv 3'-TTTTAAGCTTTCAGTCTGAGTCAGGCCCTTCT G-5'	
